# Risk factors of childhood cancer in Armenia: a case-control study

**DOI:** 10.1186/s12885-023-10565-3

**Published:** 2023-01-24

**Authors:** Manushak Avagyan, Anahit Demirchyan, Lusine Abrahamyan

**Affiliations:** 1 Turpanjian College of Health Sciences, American University of Armenia, 40 Marshal Baghramyan Ave, 0019 Yerevan, Armenia; 2 Department of Pediatric Oncology and Hematology, Yerevan State Medical University, Yerevan, Armenia; 3 Toronto General Hospital Research Institute, Toronto, ON Canada; 4 Institute for health Policy, Management and Evaluation, University of Toronto, Toronto, ON Canada

**Keywords:** Risk factors, Childhood cancer, Pregnancy, Case-control study

## Abstract

**Introduction:**

Childhood cancer (CC) is a leading cause of death among children aged 0–19 years worldwide. Each year, 400,000 new cases of CC are diagnosed globally. Given the between-country differences in CC incidence rates, types and trends, this study aimed to identify possible risk factors for CC in Armenia.

**Methods:**

We used a case-control study design and enrolled participants from the only specialized pediatric hematology and oncology center in Armenia. Cases included patients ≤ 14 years old diagnosed and treated with a malignant disease between 2017 and 2020 in the centre. Controls included patients diagnosed and treated in the center during the same period for a non-malignant disease. We conducted telephone interviews with mothers of cases and controls. Independent risk factors of cancer were identified using multivariable logistic regression analysis.

**Results:**

Overall, 234 participants (117 cases, 117 controls) were included in the study. Based on the fitted model, maternal usage of folic acid during pregnancy was protective against CC, almost twice decreasing its odds (OR = 0.54; 95% CI: 0.31–0.94). On the contrary, experiencing horrifying/terrifying event(s) during pregnancy (OR = 2.19; 95% CI: 1.18–4.07) and having induced abortions before getting pregnant with the given child (OR = 2.94; 95% CI: 1.45–5.96) were associated with higher odds for a child to develop cancer.

**Conclusion:**

Despite the limited sample size of the study, significant modifiable risk factors for CC in Armenia were identified, all of which were linked to the period of pregnancy. The data from this study adds to the limited information available from etiological CC research throughout the world, and it will increase understanding of CC risk factors in settings with small populations and low resources. Although these findings may be helpful for future research, they should be taken with caution unless validated from further larger-scale studies.

**Supplementary Information:**

The online version contains supplementary material available at 10.1186/s12885-023-10565-3.

## Background

Childhood cancer (CC) is a rare condition but it is a major cause of death among children worldwide. Each year, approximately 400,000 new cases of CC are diagnosed around the world. The leading types of CC are hematologic malignancies and brain tumors [1].

The studied risk factors that may cause or contribute to the occurrence of CC could be conditionally divided into four groups: demographic (age, sex, ethnicity), environmental/extrinsic, intrinsic (birth weight, maternal age, etc.) and genetic [2, 3]. The direct causes for CC are not yet fully identified, and the majority of the studied risk factors influence on genes causing de novo mutations, which have their unique, determined place on cancer pathogenesis [3]. Given the described uncertainty, further descriptive and molecular epidemiology studies are required to explain potential genome-environment interactions that cause CC and to develop preventative strategies.

There are considerable differences in CC incidence rates, cancer types, clinical features, and risk factors for developing CC between high-income countries and low/middle income countries (LMICs). Most probably, these differences are attributable to the different environmental, behavioral, genetic and hygienic factors, early or delayed exposure to infections and health system factors in these countries [4]. These reported differences necessitate country-specific investigation of factors related to risk factors of CC [5].

According to the report of the Armenian National Institute of Health, there were 684 total cases of newly diagnosed childhood cancers between 2008 and 2018 among patients less than 18 years old in Armenia, but this number could be underestimated given the lack of systematic reporting. According to a survey of all clinical sites that provide CC care, approximately 80–100 cases of CC are annually diagnosed in Armenia, resulting in a rate of 100–125 per million person-years [6]. These rates are comparable with the rates in the neighboring country Iran: 48–144 per million person-year [7] and in Europe, in average 137,5 per million person-years [8]. According to the estimates from recent population-based study conducted in Armenia, the cumulative incidence rate of the most common acute leukemias in children is about 15 to 19 per million child population [9]. However, based on data available from Pediatric Cancer and Blood Disorders Center of Armenia (PCBDCA), these estimates seriously underestimate the real rates [10].

So far, no study evaluated factors associated with childhood cancer in Armenia. The aim of this study was to identify possible risk factors for childhood cancer in Armenia.

## Methods

### Study design and setting

A single-center hospital-based case-control study was conducted in 2020 in the PCBDCA (located in Hematology Center (HC) after Prof. R. Yeolyan, Yerevan, Armenia). This single-center-based approach was applied, because the vast majority of pediatric cancer patients in Armenia receive their treatment in PCBDCA. The reason for this is that PCBDCA is the only center in the country that provides comprehensive diagnostic, treatment and follow-up services for children with oncological and hematological malignancies. Among few exceptions could be children with CNS tumors who may get treatment in neurological departments of non-pediatric hospitals and, hence, be omitted from the PCBDCA registry.

### Study population

Cases included patients less than 15 years of age, permanently living in Armenia and Artsakh Republic, who were diagnosed with or received care for a malignant disease at the PCBDCA during 2017–2020 and who were alive during the study time. Controls were patients less than 15 years of age, permanently living in Armenia and Artsakh Republic, who were diagnosed with or received care for a non-malignant hematologic disease at the PCBDCA during the same period (2017–2020). Sex and age were accounted for in the analysis. Mothers of cases and controls were chosen as the key informants, as they were better aware of exposures to potential risk factors of CC before and during their pregnancy and after the delivery of the child. Exclusion criteria were absence of contact information of a patient’s mother or her inability to speak Armenian.

### Study instrument and data collection

The study questionnaire was developed based on instruments used in similar studies conducted in different countries [9, 10], and the items were included after being adapted for the local specificities. The study instrument collected detailed information on demographic characteristics of the child and the parents, family’s socioeconomic characteristics and environmental exposures, child’s health and mother’s pregnancy related factors with the given child.

We attempted to contact mothers of all eligible cases and controls, and conducted telephone interviews with all those mothers available during March 2020. Telephone interview-based data collection approach was selected because of COVID-19 pandemic-related lockdown in the country.

### Study variables

The dependent variable (outcome) in this study was the presence or absence of malignancy in a child aged 0–14 years based on the confirmed diagnosis in the medical record.

Independent variables were potential risk factors of childhood cancer, including child’s demographic characteristics (age, sex, residency, diagnosis), family’s sociodemographic characteristics (e.g., residential status, family size, income), parental characteristics (e.g., age, education, employment, alcohol usage), child’s health (e.g., birth weight, birth order, breastfeeding duration, birth defects (ICD-10-CM Q00-Q99)), mother’s health and pregnancy related factors (e.g., gestational age, mode of delivery, usage of oral contraceptives, history of miscarriages, abortions, usage of folic acid, coffee, alcohol, experiencing any horrifying/terrifying events (e.g., disaster, life threatening accident, violence, sudden death of a loved one, participation in war), exposure to tobacco smoke during the pregnancy) and family’s environmental exposures (e.g., exposure to pesticides, house construction year as a proxy for possible exposure to asbestos, chemical industry or mining dump within 10 km of the house).

### Statistical analysis

The data entry and analysis were performed using IBM SPSS 23 and Stata/SE 13.0 software, respectively. The characteristics of cases and controls were summarized using descriptive statistics. Continuous variables were compared between cases and controls using t-test and categorical variables using Chi-square test or Fisher’s exact test. Variables were entered into logistic regression analysis as either dichotomous or continuous variables, while dummy variables were created for some multilevel categorical variables. All variables associated with the outcome of cancer status at p < 0.25 level of significance in descriptive comparisons [12], were included in univariate and then multivariable logistic regression analyses. Finally, a logistic regression model of independent predictors of childhood cancer was fitted. The model fit was evaluated using Hosmer-Lemeshow goodness of fit test for calibration and the area under the receiver operating characteristic (ROC) curve for discrimination [12].

## Results

Overall, the screening of the patient database identified 189 eligible cases and 212 eligible controls. Sixty-eight of the identified 189 cases and 78 of the identified 212 controls could not be contacted due to various reasons (incorrect phone number, mother unavailable at the time of data collection, or no response). Out of 121 contacted cases, completed interviews were obtained from 117 mothers and four mothers refused to participate. Of the 134 contacted controls, interviews were conducted with 117 mothers and 17 mothers refused to participate. Hence, among contacted eligible participants, the refusal rate was 3.3% for cases and 12.7% for controls.

Of the cases, 42.7% were diagnosed with acute lymphoblastic leukemia (ALL), 16.2% with lymphoma, 9.4% with different types of sarcomas, 7.7% with either central nervous system (CNS) or Wilms tumors, and the rest with germ cell tumors, histiocytosis or other malignant conditions. Of the controls, 39.3% were diagnosed with hemolytic or other anemia, 32.5% with thrombocytopenia, 9.4% with hemorrhagic vasculitis, and the rest with lymphadenopathy, coagulopathy, hemophilia or other non-malignant conditions (Supplementary Table 1).

The mean age of children was 7.1 (standard deviation (SD) 3.8) years for the cases and 5.3 (SD 3.5) years for the controls (Table 1). There were more males than females in both groups.

**Table 1:** Distribution of selected characteristics between cases with childhood cancer and controls diagnosed or treated at PCBDCA, HC, Armenia, during 2017–2020

Characteristics	Cases n = 117	Controls n = 117	Total n = 234
**Family socio-demographic characteristics**			
Living in an urban area, %(n)	65.8 (77)	67.5 (79)	66.7 (156)
Family size, mean (SD)	5.3 (1.62)	5.2 (1.57)	5.3 (2.4)
Monthly expenditures, %(n) *Less than 100 000 drams* From 100 001-300 000 drams *Above 300 000 drams*	30.2 (23) 64.5 (49) 5.2 (4)	30.4 (25) 56.1 (46) 13.4 (11)	30.4 (48) 60.1 (95) 9.5 (15)
**Child’s health**			
Child’s age (at the interview) (years), mean (SD)**	7.1 (3.79)	5.3 (3.52)	6.2 (3.66)
Sex, %(n) *Male* *Female*	57.3 (67) 42.7 (50)	62.4 (73) 37.6 (44)	59.8 (140) 40.2 (94)
Low birth weight, %(n)	7.7 (9)	12.8 (15)	10.3 (24)
Birth order, mean (SD)	1.8 (0.86)	1.8 (1.03)	1.8 (0.95)
Birth defect, %(n)	4.3 (5)	3.4 (4)	3.8 (9)
Chronic disease, %(n)	7.7 (9)	8.5 (10)	8.1 (19)
X-ray before disease diagnosis, %(n)	31.6 (37)	37.6 (44)	34.6 (81)
Breastfeeding duration (months), mean (SD)	14.0 (8.59)	14.2 (8.31)	14.1 (8.45)
**Pregnancy related factors**			
Delivery via C-section, %(n)	25.9 (30)	28.4 (33)	27.2 (63)
Folic acid during pregnancy, %(n)	38.3 (44)	53.4 (62)	45.9 (106)
Daily coffee usage during pregnancy, %(n)	68.7 (79)	58.6 (68)	63.6 (147)
Alcohol usage during pregnancy, %(n)	7.8 (9)	5.2 (6)	6.5 (15)
Number of ultrasounds during pregnancy, mean (SD)	4.7 (2.33)	5.3 (2.93)	5.0 (2.63)
Antibiotic usage during pregnancy, %(n)	10.4 (12)	6.9 (8)	8.7 (20)
Acute illness during pregnancy, %(n)	40.0 (46)	35.7 (41)	37.8 (87)
Oral contraceptive usage, %(n)	3.5 (4)	4.3 (5)	3.9 (9)
Miscarriages before getting pregnant, %(n)	21.9 (25)	21.7 (25)	21.8 (50)
Induced abortions before getting pregnant, %(n)**	30.7 (35)	12.2 (14)	21.4 (49)
Horrifying/terrifying events during the pregnancy, %(n)**	37.6 (44)	19.7 (23)	28.6 (67)
Second hand smoke exposure during pregnancy, %(n)	65.2 (75)	56.6 (65)	60.8 (140)
**Parental and family characteristics**			
MOTHER			
Mother’s age at the interview time (years), mean (SD)	33.91 (4.89)	33.24 (5.97)	33.57 (5.43)
Mother’s education, %(n) *Elementary or secondary school* *Professional technical education* *Institute/University or post-graduate*	33.6 (39) 28.4 (33) 37.9 (44)	27.4 (32) 35.0 (41) 37.6 (44)	30.5 (71) 31.8 (74) 37.8 (88)
Marital status, %(n) *Married* *Other*	93.2 (109) 6.8 (8)	96.6 (113) 3.4 (4)	94.9 (222) 5.1 (12)
Ever cigarette smoking, %(n)	2.6 (3)	5.1 (6)	3.8 (9)
Current cigarette smoking, %(n)	0.0 (0)	0.9 (1)	0.45 (1)
Never using alcohol, %(n)	47.0 (55)	56.0 (65)	51.5 (120)
FATHER			
Father’s age at the time of the interview (years), mean (SD)	38.6 (6.23)	37.8 (7.85)	38.2 (7.04)
Father’s education, %(n) *Elementary or secondary school* *Professional technical education* *Institute/University or post-graduate*	47.9 (56) 20.5 (24) 31.6 (37)	41.0 (48) 19.7 (23) 39.3 (46)	44.4 (104) 20.1 (47) 35.5 (83)
Father’s ever smoking, %(n)	83.6 (97)	76.7 (89)	80.2 (186)
Current smoking, %(n)	62.8 (75)	67.5 (79)	65.5 (154)
FAMILY MEMBERS			
Daily second-hand smoke exposure, %(n)	21.4 (25)	17.9 (21)	19.7 (46)
Binge drinking in family, %(n)	6.8 (8)	6.0 (7)	6.4 (15)
Core family member with diagnosed cancer, %(n)	34.2 (40)	23.1 (27)	28.6 (67)
**Family environmental exposures**			
Pesticide usage, %(n)	21.6 (25)	18.4 (21)	20.0 (46)
Child’s presence during pesticide sprays, %(n)	5.12 (6)	1.8 (2)	3.33 (8)
Average age of the house (years), mean (SD)	35.9 (16.07)	32.8 (18.15)	34.4 (17.11)
Housing conditions, %(n) *Good* *Average (meets basic needs)* *Poor*	32.5 (38) 61.5 (72) 6.0 (7)	26.1 (30) 67.8 (78) 6.1 (7)	29.3 (68) 64.7 (150) 6.0 (14)
Chemical industries within 10 km from the house, %(n)	13.7 (16)	12.2 (14)	12.9 (30)
Mining dump within 10 km from the house, %(n)	7.7 (9)	14.8 (17)	11.2 (26)

SD = standard deviation

**p-value < 0.01

p-values were adjusted for age and sex

In comparisons adjusted for age and sex, a statistically significant difference was detected between cases and controls regarding their mother’s history of induced abortions before getting pregnant with the given child and significantly higher proportion of mothers of cases reported experiencing highly stressful event(s) during the pregnancy.

The analysis comparing solely ALL cases with the controls revealed significant findings regarding highly stressful events during pregnancy, and mothers of leukemia patients reported experiencing very stressful situations during pregnancy at a significantly higher proportions than mothers of controls (OR = 2.50; 95% CI: 1.18–5.28). The rest of the variables yielded insignificant results, perhaps, due to insufficient power of the study because of the small number of cases with ALL (n = 50).

Table 2 depicts the final fitted model of independent predictors of CC with three significant risk factors. According to this model, mother’s taking folic acid during pregnancy was associated with 46% lower odds of having a child with cancer compared to not taking folic acid during pregnancy (OR = 0.54; 95% CI: 0.31–0.94). The history of induced abortions before getting pregnant with the given child was associated with 2.94 times higher odds for the child to develop cancer (95% CI: 1.45–5.96). The odds of developing cancer by a child, whose mother reported experiencing a horrifying/terrifying event during pregnancy was 2.19 (95% CI: 1.18–4.07) times the odds of developing cancer by a child, whose mother reported no such experience. The p-value of the Hosmer-Lemeshow goodness-of-fit test for the final model was 0.615, indicating a good fit (acceptable level of calibration) and the area under the ROC curve was 0.699 indicating acceptable discrimination.

**Table 2:** Multiple logistic regression model of determinants of childhood cancer among patients treated at PCBDCA, HC, Armenia, during 2017–2020 (n = 229)

Characteristics	Odds Ratio	95% CI	P-value
Folic acid usage during pregnancy	0.54	0.31–0.94	0.030
Induced abortions before getting pregnant	2.94	1.45–5.96	0.003
Horrifying/terrifying events during the pregnancy	2.19	1.18–4.07	0.013
Family member diagnosed with cancer	1.71	0.93–3.18	0.085

Model fit statistics: *Hosmer-Lemeshow goodness of fit test, p = 0.615;*

*Area under the receiver operating characteristics curve: 0.699; McFadden’s R squared: 0.0847*

## Discussion

Current study aimed to reveal independent risk factors of CC among children aged 0–14 years old in Armenia, thus filling an important research gap in this area. The study identified three important predictors of CC: maternal folic acid usage during pregnancy as a protective factor and two strong risk factors of CC—history of induced abortions before the pregnancy and trauma-induced stress during the pregnancy. These findings are important for planning new research initiatives. However, unless validated from further larger-scale studies, they should be taken with prudence.

Similar case-control studies in other countries had comparable study populations in terms of patients’ sex distribution with male predominance, and cancer type distribution with leukemias, lymphomas and brain tumors being the most common types of CC [11, 12]. Similarly, CC cases were more commonly living in urban areas in other studies as well, and the maternal age range in this study was comparable to those in other case-control studies [12, 13].

The protective effect of maternal folic acid usage before and during pregnancy on the risk of development of childhood cancer identified in this study was consistent with the literature, as many studies found that taking folic acid before and during pregnancy was significantly inversely associated with the cancer risk (leukemia, brain tumor) in a child [14]. Indeed, several studies from the United States and Canada recorded a decrease in childhood cancer incidence (neuroblastoma, Wilms tumor and primitive neuroectodermal tumors (PNET)) after folic acid fortification of food [15]. Folate is considered as an essential nutrient for the cell multiplication (as a coenzyme for DNA synthesis) and cell homeostasis (metabolism and regeneration) [16]. According to WHO standards for maternal and neonatal care, mothers should start taking 400 µg folic acid daily two months before getting pregnant and continue taking it until the 12th week of pregnancy [17]. This study adds to the existing evidence on the importance of following this recommendation.

According to this study findings, the history of induced abortions before pregnancy increased the risk of childhood cancer almost threefold. This finding is consistent with the findings of a number of studies indicating that the past history of fetal losses including induced abortions, miscarriages and stillbirths is associated with a higher incidence of cancer (leukemia, neuroblastoma, soft tissue sarcoma) in children [16, 17]. However, some studies report lack of such association or insignificant associations [18, 19]. A matched case-control study conducted by Children’s Cancer Group and the Pediatric Oncology Group identified two times higher risk of neuroblastoma in children whose mothers had a history of two or more previous induced abortions [19].

The history of maternal trauma-induced stress during pregnancy was a risk factor for developing childhood cancer in the offspring in this study. A population-based cohort study conducted in Denmark and Sweden that included 39,002 children born to women who had psychological stress (parental death) during pregnancy, identified a similar link between this experience and the risk of cancer in the offspring. In particular, the risk was increased for leukemia (standardized incidence ratio (SIR), 1.49; P = 0.004), testicular cancer (SIR, 1.80; P = 0.02) and colon cancer (SIR, 3.95; P = 0.003) before the age of 15 years [20]. There are studies indicating the role of the stress during pregnancy for increasing the risk of childhood cancer [21]. There is a proposed hypothesis explaining the pathogenesis of a stress-induced cancer by the mediated inhibition of the enzyme, which initiates activation of cancerous cells to get under immune surveillance, leading to the newly generated malignant cells to continue growing without being checked by the immune system [20]. This mechanisms could explain the findings of a cohort study conducted in Israel, which observed higher cancer incidence (for leukemia, lymphoma and melanoma) among bereaved Jewish people [22]. However, the mechanism underlying the association between maternal stress during pregnancy and carcinogenesis in the offspring is not yet clearly understood.

This is the first study that evaluated risk factors of childhood cancers in Armenia. Both cases and controls were selected from the same hospital, which is the only place in the county where children with hematologic and oncologic diseases can get specialized diagnosis and treatment. While this could indicate that cases and controls potentially experienced similar exposures, we cannot generalize the findings to overall Armenian population considering the selection of our control population (i.e., ideal controls would have been healthy children with similar exposures and characteristics).This study did not identify significant associations between child’s second-hand smoking and childhood cancer, or other well-known environmental exposures confirmed as risk factors for childhood cancer, such as pesticides, chemicals, radiation or others. The underlying reason for this could be the small size of the studied sample, which was the major limitation of the study overall and the wide diversity of diseases with potentially different sets of risk factors combined in a heterogeneous group of CC. Recall bias could be a potential limitation of this study due to long interval between the onset of the child’s disease and the interview. To minimize this bias, we selected participants who were diagnosed or received CC treatment relatively recently.

The telephone interviews were conducted by six trained interviewers; however, they were aware of the participant’s case and control status which could introduce an interviewer bias. There was also a likelihood that mothers of cases might remember some events, which mothers of controls might disregard or had forgotten.

Selection bias could be a potential threat to the generalizability of the study findings, as patients with missing contact information or being not registered in PCBDCA (e.g., patients with CNS tumors) could somehow differ from the participants with the contact information available, although it is unlikely that the difference, if existing, could seriously alter the study findings. Cases of CNS malignancy could be underrepresented considering the fact that these are treated mostly in surgical departments of other hospitals after which patients are not referred to a pediatric oncology clinic. Additionally, it is a worldwide difficulty for population-based cancer registries to approve full coverage, particularly for the most prevalent non-malignant CNS tumors in children, which are eligible for registration but are often overlooked by the system[23]. Furthermore, twenty-three patients diagnosed with cancer in the PCBDCA during the same period (2017–2020) who did not survive by the time of the study were excluded from the survey because of ethical considerations of conducting interviews with their mothers. This could have influenced the study findings if the non-survivors were different than the study cases. However, the analysis of the available characteristics of the non-survivors demonstrated that they did not differ from the survivors in terms of age, sex, residency, and the cancer type. Inclusion of participants with different types of cancer as cases is another limitation of this study, as there are cancer-specific risk factors, which cannot be identified with this approach.

A number of important recommendations could be derived from the findings of this study. First, they add another important argument for folic acid supplementation before and during pregnancy, which is preventing CC. Although folic acid supplementation of pregnant women is currently recommended by the Ministry of Health of Armenia to prevent neural tube defects, there are issues with its timing and coverage [24]. Hence, efforts should be undertaken to achieve timely and universal coverage of all pregnant women with this supplementation. Also, this study highlights the need to increase the awareness among women about the importance of using safe and effective methods of birth control and family planning to avoid induced abortions. Education of reproductive age women and their family members on the importance of stress reduction during pregnancy is another important area for improvement. To investigate childhood cancer-specific risk factors, large-scale studies with a larger sample size of children with homogeneous group of CC are recommended.

## Electronic supplementary material

Below is the link to the electronic supplementary material.

Supplementary Material 1

## Data Availability

The datasets generated and analysed during the current study are available from the corresponding author on reasonable request.

## Abbreviations

ALL: Acute lymphoblastic leukemia
AUC: Area under curve
CC: Childhood cancer
CI: Confidence interval
CNS: Central nervous system
HC: Hematology Center
LMIC: Low- and middle- income country
OR: Odds ratio
PCBDCA: Pediatric Cancer and Blood Disorders Center of Armenia
ROC: Receiver operating characteristic
SD: Standard deviation
WHO: World Health Organization

## References

[1] Lam CG, Howard SC, Bouffet E, Pritchard-Jones K. “Science and health for all children with cancer,” *Science (80-.)*, vol. 363, no. 6432, pp. 1182–1186, Mar. 2019. 10.1126/science.aaw4892 30872518

[2] Lunde A, Melve KK, Gjessing HK, Skjaerven R, Irgens LM. “Genetic and Environmental Influences on Birth Weight, Birth Length, Head Circumference, and Gestational Age by Use of Population-based Parent-Offspring Data,” *Am. J. Epidemiol*, vol. 165, no. 7, pp. 734–741, Feb. 2007. 10.1093/aje/kwk107 17311798

[3] Whelan K, Alva E. Epidemiology of Childhood Cancer. Elsevier Inc.; 2018.

[4] Bhakta N et al. “Childhood cancer burden: a review of global estimates,” *The Lancet Oncology*, vol. 20, no. 1. Lancet Publishing Group, pp. e42–e53, 01-Jan-2019. 10.1016/S1470-2045(18)30761-7 30614477

[5] Rodriguez-Galindo C, Friedrich P, Morrissey L, Frazier L. “Global challenges in pediatric oncology,”Current Opinion in Pediatrics, vol. 25, no. 1. pp.3–15, Feb-2013. 10.1097/MOP.0b013e32835c1cbe 23295716

[6] Hovhannisyan S et al. “Pediatric cancer care in Armenia: The results of a qualitative analysis.,” 10.1200/JCO.2020.38.15_suppl.e19002, vol. 38, no. 15_suppl, pp. e19002–e19002, May 2020

[7] Mousavi S, Pourfeizi A, Dastgiri S. “Childhood cancer in Iran,” *J. Pediatr. Hematol. Oncol*, vol. 32, no. 5, pp. 376–382, Jul. 2010. 10.1097/MPH.0b013e3181e003f7 20588194

[8] Steliarova-Foucher E et al. “Changing geographical patterns and trends in cancer incidence in children and adolescents in Europe, 1991–2010 (Automated Childhood Cancer Information System): a population-based study,” *Lancet Oncol*, vol. 19, no. 9, pp. 1159–1169, Sep. 2018. 10.1016/S1470-2045(18)30423-6 PMC6120055 30098952

[9] Khondkaryan L et al. “Incidence and Risk Factors of Acute Leukemias in Armenia: A Population-Based Study,” *Asian Pac. J. Cancer Prev*, vol. 23, no. 11, pp. 3869–3875, Nov. 2022. 10.31557/APJCP.2022.23.11.3869 PMC9930974 36444600

[10] Krmoyan L et al. “Childhood Acute Leukemias in Armenia,” *Clin. Lymphoma Myeloma Leuk*, vol. 19, p. S195, Sep. 2019.

[11] Abelyan G, Abrahamyan L, Yenokyan G. A case-control study of risk factors of chronic venous ulceration in patients with varicose veins. Phlebology. Feb. 2018;33(1):60–7. 10.1177/0268355516687677 28077026

[12] Hosmer DW, Lemeshow S. “Applied Logistic Regression,” *Appl. Logist. Regres*, Jan. 2005.

[13] Ezzat S et al. “Environmental, maternal, and reproductive risk factors for childhood acute lymphoblastic leukemia in Egypt: a case-control study,” *BMC Cancer* 2016 161, vol. 16, no. 1, pp. 1–7, Aug. 2016. 10.1186/s12885-016-2689-z PMC4992254 27544685

[14] Wan Ismail WR, Abdul Rahman R, Rahman NAA, Atil A, Nawi AM. The protective effect of maternal folic acid supplementation on Childhood Cancer: a systematic review and Meta-analysis of case-control studies. J Prev Med Public Heal. Jul. 2019;52(4):205–13. 10.3961/jpmph.19.020 PMC6686110 31390683

[15] Grupp SG et al. “Pediatric cancer rates after universal folic acid flour fortification in ontario,” *J. Clin. Pharmacol*, vol. 51, no. 1, pp. 60–65, Jan. 2011. 10.1177/0091270010365553 20457589

[16] Stover PJ. “ Physiology of Folate and Vitamin B 12 in Health and Disease,” *Nutr. Rev*, vol. 62, pp. S3–S12, Jun. 2004. 10.1111/j.1753-4887.2004.tb00070.x 15298442

[17] *WHO | Guideline Daily iron and folic acid supplementation in pregnant women*. 2012. 23586119

[18] Ou Shu X (2002). Birth characteristics, maternal reproductive history, hormone use during pregnancy, and risk of childhood acute lymphocytic leukemia by immunophenotype (United States). Cancer Causes Control.

[19] Hamrick SEG, Olshan AF, Neglia JP, Pollock BH. “Association of pregnancy history and birth characteristics with neuroblastoma: a report from the Children’s Cancer Group and the Pediatric Oncology Group,” *Paediatr. Perinat. Epidemiol*, vol. 15, no. 4, pp. 328–337, Oct. 2001. 10.1046/j.1365-3016.2001.0376a.x 11703680

[20] Bermejo JL, Sundquist J, Hemminki K. “Risk of Cancer among the Offspring of Women Who Experienced Parental Death during Pregnancy,” *Cancer Epidemiol Biomarkers Prev*, vol. 16, no. 11, pp. 2204–2206, Nov. 2007. 10.1158/1055-9965.EPI-07-0638 18006907

[21] Li J et al. “Antenatal maternal bereavement and childhood cancer in the offspring: A population-based cohort study in 6 million children,” *Br. J. Cancer*, vol. 107, no. 3, pp. 544–548, Jul. 2012. 10.1038/bjc.2012.288 PMC3405225 22759879

[22] Levav I, Kohn R, Iscovich J, Abramson JH, Tsai WY, Vigdorovich D (2000). Cancer incidence and survival following bereavement. Am J Public Health.

[23] Stiller CA, Bayne AM, Chakrabarty A, Kenny T, Chumas P. Incidence of childhood CNS tumours in Britain and variation in rates by definition of malignant behaviour: Population-based study. BMC Cancer. Feb. 2019;19(1):1–15. 10.1186/s12885-019-5344-7 PMC6371471 30744596

[24] Demirchyan PV, Melkom Melkomian A, Mnatsakanyan D. K, “Formative Research on Infant and Young Child Health and Nutrition in Armenia,” 2015.

