# Supplementary material for: Risk factors of childhood cancer in Armenia: a case-control study
Source: BMC Cancer. 2023 Jan 24;23:81. doi: 10.1186/s12885-023-10565-3 (PMC9873390; doi:10.1186/s12885-023-10565-3)
Supplement: Supplementary file 1 — Supplementary Material 1 [file 12885_2023_10565_MOESM1_ESM.docx]

## **Supplementary Table 1.**

## **Primary diagnosis in children in the control group**

| Diagnosis | n = 117 |
| --- | --- |
| Acquired hemolytic anemia, unspecified | 24 |
| Acute lymphadenitis, unspecified | 6 |
| Atopic dermatitis, unspecified | 1 |
| Coagulopathy, unspecified | 5 |
| Iron deficiency anemia | 15 |
| Hemophilia A | 4 |
| Hereditary spherocytosis | 1 |
| Immune thrombocytopenic purpura | 38 |
| Leukemoid reaction | 1 |
| Leukocytopenia, unspecified | 1 |
| Pulmonary embolism | 1 |
| Splenomegaly, not elsewhere classified | 2 |
| Vasculitis limited to skin, not elsewhere classified | 11 |
| Vitamin B12 deficiency anemia | 7 |
